# Supplementary material for: A Multi-Omics Characterization of the Natural Product Potential of Tropical Filamentous Marine Cyanobacteria
Source: Mar Drugs. 2021 Jan 6;19(1):20. doi: 10.3390/md19010020 (PMC7825088; doi:10.3390/md19010020)
Supplement: Supplementary file 1 [file marinedrugs-19-00020-s001.zip › marinedrugs-1036488 SI/Figure S1-S4.pdf]

marine drugs

MDPI

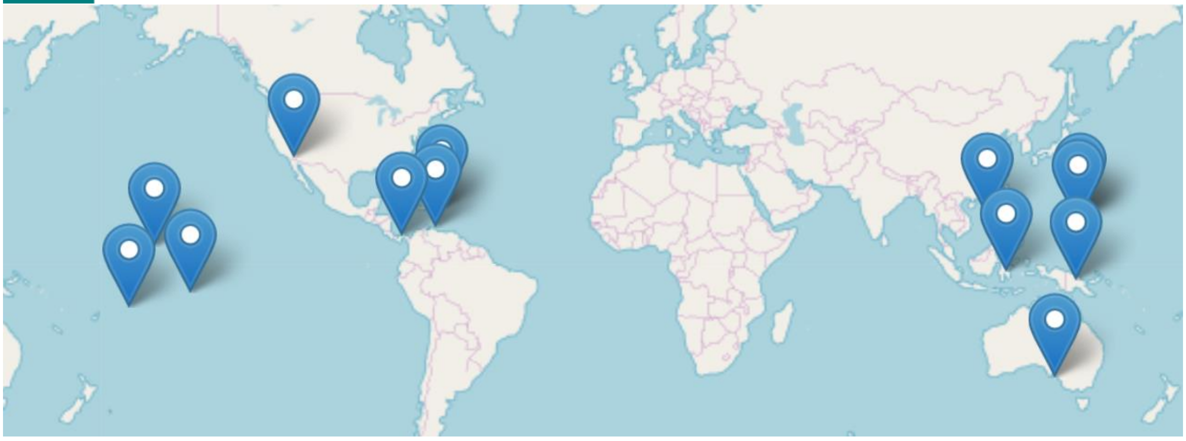

Figure S1. Geographic locations for 165 collected metagenomic marine cyanobacterial samples. Locations include American Samoa, Guam, Panama, Curaçao, Papua New Guinea, Indonesia, Marion Bay, Millennium Atoll, Palmyra Atoll, Puerto Rico, Saipan, Salton Sea, South China Sea (more details on Dataset S1, sheet 1).

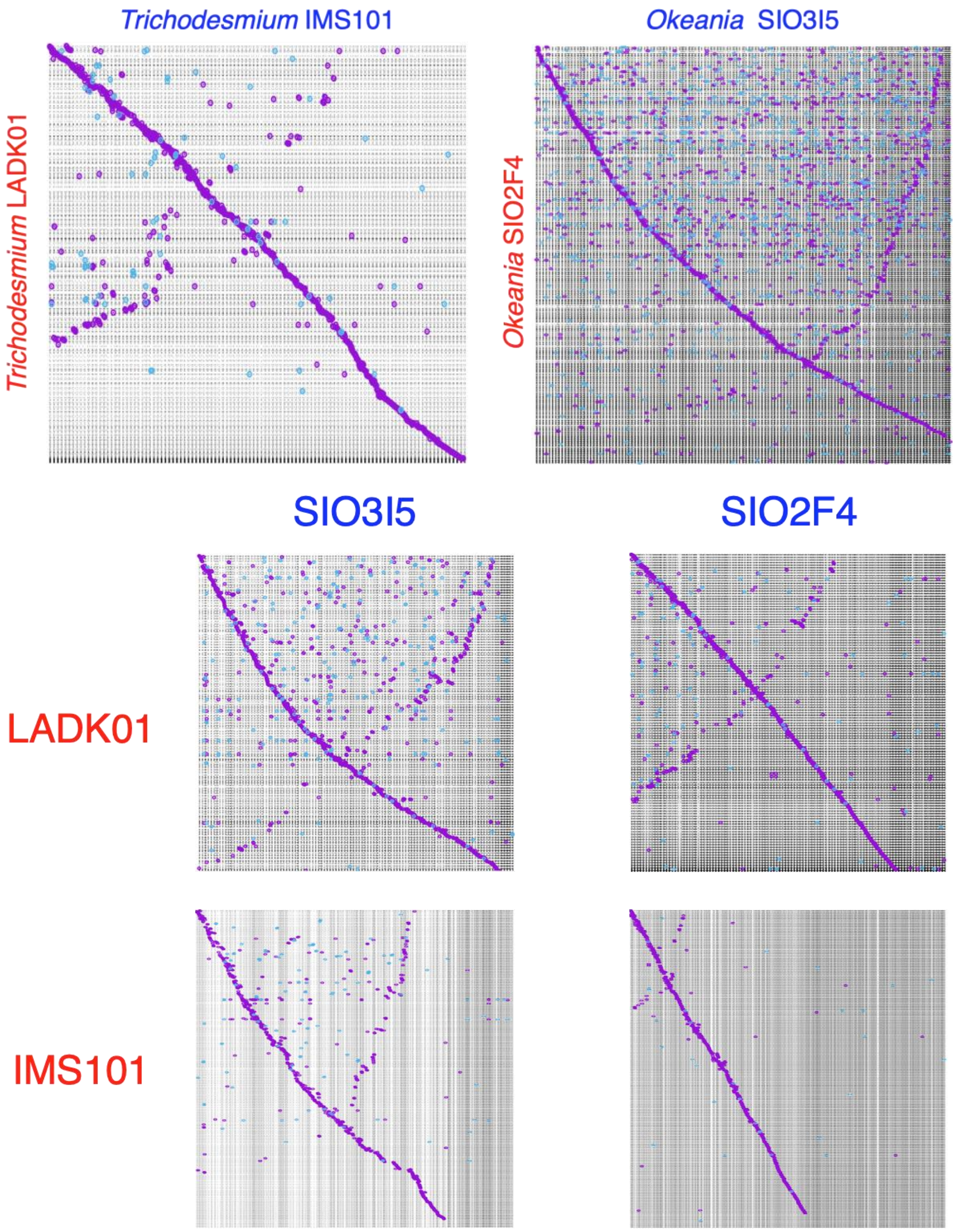

Figure S2. MUMmer genome alignments between two *Okeania* (SIO2F4 and SIO3I5) and two *Trichodesmium* (IMS101 and LADK01) samples. All plots display a linear trend between the pair of genomes, indicating good synteny between the four strains.

a)

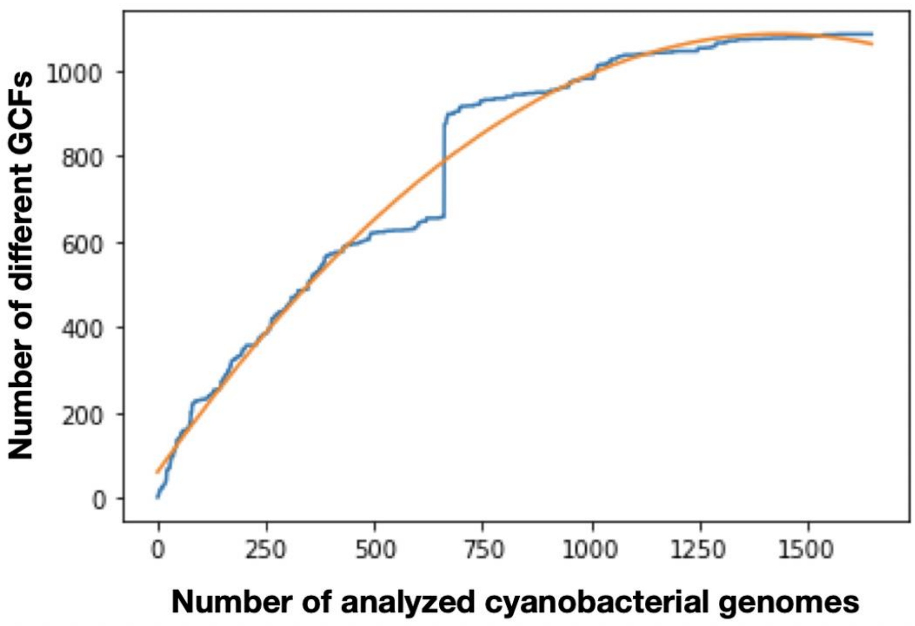

b)

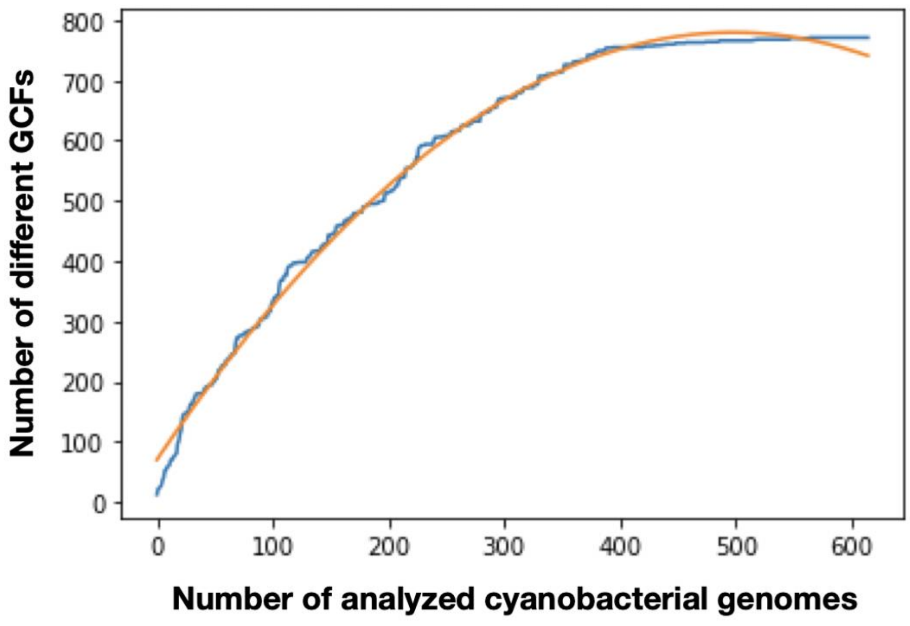

Figure S3. Rarefaction curve from the presence/absence of GCFs. Actual counts are represented in the blue line and fitted curve is indicated in orange. Both curves (a for all investigated cyanobacterial genomes and b for all cyanobacterial except *Prochlorococcus* and *Synechococcus* genomes) indicate that only a few hundred more cyanobacterial genomes are necessary to be sequenced to reach the total BGC diversity.

CCMSLIB00005721586
“Clerodane diterpenoid”

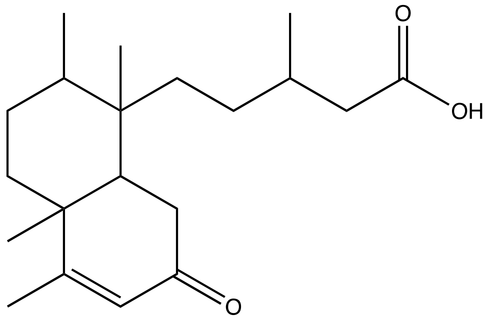

CCMSLIB00000084870
“Chromone”

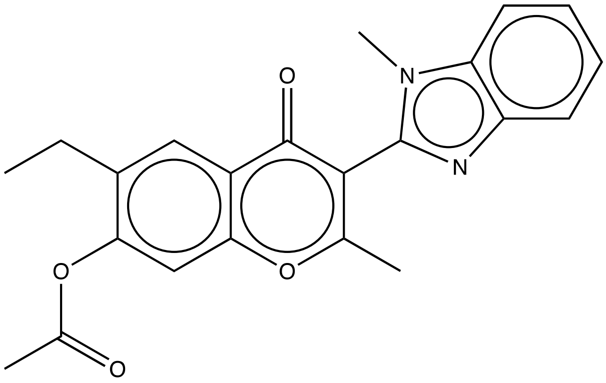

CCMSLIB00000853386
“Ingenane diterpenoid”

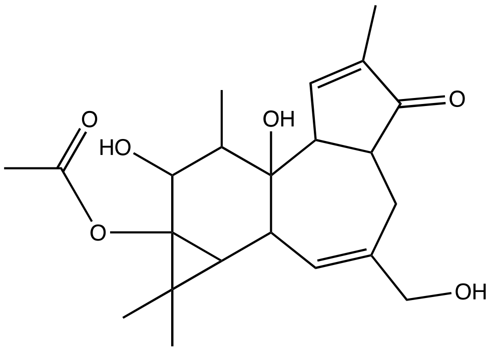

CCMSLIB0000546483
“Dihydroxy bile acid”

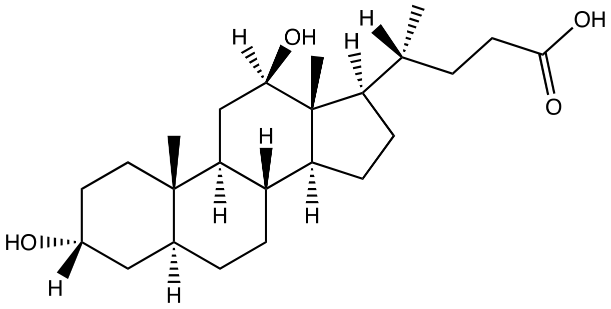

CCMSLIB00000847331
“8-O-methylated flavonoid”

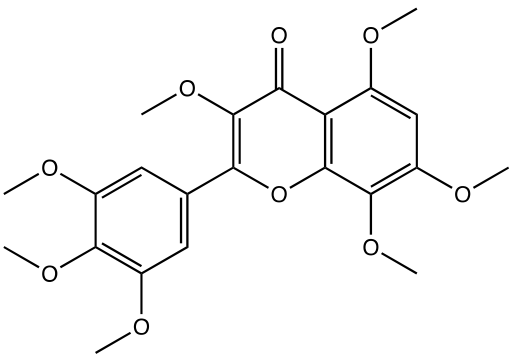

CCMSLIB00004713996
“Medium-chain fatty acid”

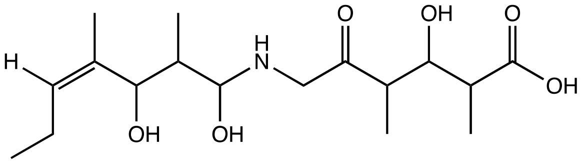

Figure S4. Predicted structures for molecules named after their most detailed molecular classes (complete IUPAC name is detailed in Dataset S1, sheet 2, according to their CCMS code).
